# Peer Review of “Finding Potential Adverse Events in the Unstructured Text of Electronic Health Care Records: Development of the Shakespeare Method”

**DOI:** 10.2196/31548

**Published:** 2021-08-11

*This is a peer-review report submitted for the paper “Finding Potential Adverse Events in the Unstructured Text of Electronic Health Care Records: Development of the Shakespeare Method”*

## Round 1 Review

### General Comments

This paper [1] described the “Shakespeare method,” which was designed to discover associations between adverse events (AEs) caused by blood transfusion from unstructured electronic health record (EHR) notes. The authors applied this method on the MIMIC-III data set and seemed to be able to find transfusion AEs (TAEs) and potential TAEs (PTAEs) that were unknown when those EHR notes were developed.

### Specific Comments

#### Major Comments

1. Is there any plan to release all the code/scripts used in this study? The method seems to be complex involving multiple steps; it will be very difficult to reproduce the results if the code is not available.
2. The manuscript should include more details on how the transfusion and comparison groups were created.
3. The author mentioned that the latent Dirichlet allocation (LDA) method they used in topic modeling requires the number of topics to be selected a priori. In this study, they set it to 45. Some questions:
  - How robust is the “Shakespeare method” with respect to this value? If a different value is chosen, will the method find similar topics? Similar notes for manual document review? Similar TAEs/PTAEs?
  - How would you determine this value if the method is applied to detect AEs for other treatments?
  - A brief introduction to the LDA method should be included in the manuscript.
4. In the *Results* section, the authors mentioned “Despite the inclusion of 1 to 5 grams in the vectorization, the terms that we extracted during classification were unigrams.” That seems to be quite a coincidence; is there any explanation? If only unigrams are used in the bag-of-word representation, will the results be different? Does it mean only unigrams are needed in the future application of this method?
5. If possible, applying the method in other data sets or for other types of treatment will help to understand how generalizable the method is.
6. On page 4, section *The Shakespeare Method*: “Trim the n-gram vectors in the target group to those that are significant for the target group.” How is the trimming performed? How important is it for the final result?

#### Minor Comments

1. In the *Abstract* section, the authors wrote “We chose the case of transfusion adverse events (TAEs) and potential TAEs (PTAEs) because real dates were obscured in the study data, and new TAE types were becoming recognized during the study data period.” The causal relationship here is a little confusing.
2. On page 3, the authors wrote, “The Shakespeare method has three parts,” but the following bullet-point list has 5 items.
3. On page 8: “The Shakespeare method would likely generalize to other her notes and possibly other types of medical texts.” An additional “her” is inserted.

## Round 2 Review

The revision addressed my previous concerns. I have no further comments.

## Abbreviations

AE: adverse event
EHR: electronic health record
LDA: latent Dirichlet allocation
PTAE: potential transfusion adverse event
TAE: transfusion adverse event
